# A Combined AFM and Lateral Stretch Device Enables Microindentation Analyses of Living Cells at High Strains

**DOI:** 10.3390/mps2020043

**Published:** 2019-05-24

**Authors:** Dave Ahrens, Wolfgang Rubner, Ronald Springer, Nico Hampe, Jenny Gehlen, Thomas M. Magin, Bernd Hoffmann, Rudolf Merkel

**Affiliations:** 1 Institute of Complex Systems: Biomechanics (ICS-7), Forschungszentrum Jülich, 52428 Jülich, Germany; d.ahrens@fz-juelich.de (D.A.); w.rubner@web.de (W.R.); r.springer@fz-juelich.de (R.S.); n.hampe@fz-juelich.de (N.H.); jenny.gehlen@stud.ki.se (J.G.); b.hoffmann@fz-juelich.de (B.H.); 2 Institute of Biology, Division of Cell and Developmental Biology, University of Leipzig, 04103 Leipzig, Germany; thomas.magin@uni-leipzig.de

**Keywords:** cell mechanics, cell stretching, atomic force microscopy, strain stiffening, cytokeratin network mechanics

## Abstract

Mechanical characterization of living cells undergoing substantial external strain promises insights into material properties and functional principles of mechanically active tissues. However, due to the high strains that occur in physiological situations (up to 50%) and the complex structure of living cells, suitable experimental techniques are rare. In this study, we introduce a new system composed of an atomic force microscope (AFM), a cell stretching system based on elastomeric substrates, and light microscopy. With this system, we investigated the influence of mechanical stretch on monolayers of keratinocytes. In repeated indentations at the same location on one particular cell, we found significant stiffening at 25% and 50% strain amplitude. To study the contribution of intermediate filaments, we used a mutant keratinocyte cell line devoid of all keratins. For those cells, we found a softening in comparison to the wild type, which was even more pronounced at higher strain amplitudes.

## 1. Introduction

Living cells exhibit fascinating mechanical properties that are decisive for many fundamental processes like tissue maintenance, wound healing, or the resilience of skin against external forces. In all such mechanical processes, the three filamentous cytoskeletal systems—actin filaments (AFs), microtubules (MT), and intermediate filaments (IFs)—interact synergistically to build and control a fascinating composite material with unique mechanical properties.

Experiments using diverse force probes like atomic force microscope (AFM) cantilevers [1,2], optical tweezers [3,4,5,6], magnetic tweezers [7,8,9], or micropipette aspiration [10,11], were instrumental in elucidating complex biomaterial properties like strain stiffening [12,13,14], glass-like behavior [15,16], or stretch-induced fluidization [17] of cells. In a second line of experiments, active responses of cells to external stretch were investigated using freely suspended sheets of endothelial cells [18] or cells plated on highly stretchable substrates [19,20,21,22]. In all cases, the experiments suggested that the mechanical properties of living cells change dramatically upon external stretch. 

Measurements of mechanical properties of cells subject to external stretch are substantially complicated due to high cell-to-cell variability and extreme spatial variations, even at the single cell level. Moreover, applying physiological strain amplitudes of up to 50% [23,24] during micro- or nanomechanical tests is challenging. To enable reliable measurements in such conditions, we built an instrument that combined continuous light microscopy with atomic force microscopy and large amplitude stretching via elastomeric substrates. Because the epidermis is a mechanically very active organ, we chose its main cellular component, the keratinocytes, for these prototype experiments.

A second focus is on keratins and their role in the mechanical resilience of epidermal cell monolayers against high strains [25]. Keratins form the largest subgroup of IFs with 54 members expressed in epithelial tissues and contribute significantly to the stiffness of cells against indentation [26,27]. Furthermore, in contrast to AFs and MTs, IFs are extremely stretchable. In AFM experiments, reconstituted IFs could be stretched to 260% [28] and 350% [29] without rupturing. Moreover, as intracellular components in confluent monolayers, IFs resisted strains of more than 100% [30]. In reconstituted networks, both their stiffness and their resilience against mechanical strain correlate with the applied level of deformation [12]. 

With regard to stress resilience, IFs and AFs mechanically complement each other. For small strains, AFs form networks that are highly tensed, while IFs remain floppy. Beyond a critical point of strain (somewhere above 20%), reconstituted AFs rupture. This strain level corresponds roughly to the critical strain amplitude where solutions of intermediate filaments start to stiffen [12]. We hypothesize that this behavior also persists in living cell monolayers. Therefore, we performed comparative measurements of a keratin-free keratinocyte cell line (knockout, KO) and their wild-type control (WT), both grown as confluent cell monolayers. 

In the first part of this work, we give a detailed characterization of our system. This is followed by biomechanical analyses of keratinocyte monolayers under large strain in which we found compelling evidence of keratins being responsible for cell stiffening at large strains.

## 2. Materials and Methods

### 2.1. Preparation of Cell Chambers

Molding of elastomeric cell chambers was based on Faust et al. [22]. The same chamber geometry and molds were used. An addition-curing polydimethylsiloxane (PDMS) elastomer was prepared from a two-component formulation (Silpuran 2430 A/B, Wacker Chemie AG, München, Germany). Part A and B were mixed in a ratio of 1 to 4 by weight. Preparation was performed in a cold room at 6 °C, while curing was achieved overnight at room temperature. Otherwise, the preparation was done as described previously [22]. The silicone rubber exhibited a Young’s modulus of ~370 kPa after crosslinking. For a better mimic of physiological conditions, the chamber bottoms were covered by 130 µm of a softer PDMS elastomer (50 kPa; Sylgard 184, Dow Corning GmbH, Wiesbaden, Germany) via spin coating. Coated chambers were cured again for 16 h at 60 °C. Elastomer stiffness was calibrated as described in the supplement of [31]. In brief, elastomer layers with defined thickness were prepared on rigid support and indented with a flat cylindrical punch attached to a force sensor. Indentation was done in several steps of 200 µm, each followed by a 40 min relaxation period. Because the ratio of equilibrium force to indentation depth is directly proportional to the Young’s modulus, this yielded the latter. The necessary proportionality factor was determined on layers of stiffer silicone rubber from which cylindrical test pieces could be prepared and calibrated by vertical stretching as described in [32]. 

### 2.2. Preparation of Calibration Samples

For calibration of local strains, fluorescent beads were immobilized on top of the soft elastomer layer by the following method. PDMS surfaces were functionalized with (3-aminopropyl)triethoxysilane (APTES, Sigma-Aldrich Chemie GmbH, München, Germany). To this end, they were incubated for 3 min with a freshly prepared 5% solution of APTES (in 5% water in ethanol, pH 4.5–5.5 with acetic acid, age of solution 1.5–2 h), washed with ethanol, and dried in vacuo. Fluorescent beads (FluoSpheres, carboxylate-modified, 0.2 µm, blue, Thermo Fisher Scientific, Waltham, MA, USA) were activated by 1-ethyl-3-(3-dimethylaminopropyl) carbodiimide hydrochloride (EDC; Sigma-Aldrich Chemie GmbH, München, Germany) and N-hydroxysulfosuccinimide (NHS; Sigma-Aldrich Chemie GmbH, München, Germany) via 15 min incubation in MES buffer (2-(N-morpholino)ethanesulfonic acid; 50 mM, pH 6.0 with NaOH) containing 0.1% sodium dodecyl sulfate, 0.6 ppm beads, 20 mg/mL EDC, and 20 mg/mL NHS. Silanized substrates were incubated for 1 min with activated beads and thoroughly rinsed with water.

For thickness determination of the soft PDMS layer on top of the stiffer chamber bottom, fluorescent beads were deposited on the latter before overlaying it with soft elastomer. To this end, bead suspensions (PSI-B 0.5 fluorescent blue (354/450) plain surface, Kisker Biotech, Steinfurt, Germany) were diluted with pure ethanol (AnalaR Normapur, VWR International, Darmstadt, Germany) at a ratio of 1:100, pipetted on uncoated Silpuran chamber bottoms, and evenly spread. After evaporation, the second silicone and the second bead layer were prepared on top as described above. Thickness was determined from the distance between the focus planes of both bead layers in fluorescence microscopy. The necessary correction for refractive index mismatch was applied.

### 2.3. Stretching Setup and Protocol

Cell stretcher and chamber holders were based on the design described in Faust et al. [22]. Major alterations due to space limitations below the AFM head (Nanowizard 1, JPK, Berlin, Germany) are described in the results section. Chambers were stretched by 7.5% of their size (corresponding to 1.5 mm) before experiments to avoid sagging of the chamber bottom. Strain was increased at a constant speed of 0.75%/s (0.15 mm/s) until the desired target strain was reached, and the system was arrested. Target strains were 25% and 50%, and the measurement period at each strain was 30 min. Due to the design of the chamber and the stretcher, the strain was uniaxial.

### 2.4. Strain Calibration

All strain amplitudes given in the text refer to externally applied strains by the stepper motor. Local strains were calibrated by stretching an empty silicone chamber coupled with fluorescent beads as markers for local in-plane strains of the chamber surface. Rectangular template regions (33 µm by 263 µm) containing approximately 100 beads were selected. Normalized cross-correlation was used to find the templates again at different strain amplitudes. The affine transformation that best morphed the undeformed template into the stretched one was calculated by the Lucas–Kanade algorithm [33]. Via this transformation, strained template regions could be compared with the unstretched state, and local strains could be determined.

### 2.5. Atomic Force Microscopy

Indentations on cells were performed using an AFM equipped with a cantilever of nominal resonance frequency f_0_ = 6 kHz and a spring constant k = 0.03 N/m (Arrow-TL1-50, NanoWorld AG, Neuchatel, Switzerland). A spherical silica bead (radius: 3.6 µm, Kisker Biotech, PSI-5.0, surface plain, Steinfurt, Germany) was glued (UHU plus Endfest 300; UHU, Bühl, Baden, Germany) onto the cantilever tip as previously described [26]. AFM indentations were performed at room temperature with a cantilever speed of 0.5 µm/s for approach and 2 µm/s for retract. Indentations were repeated six times for each position with 2 s pauses between measurements. A sampling rate of 2 kHz and a force set point of 1.5 nN were used. Before each measurement, cantilever spring constants and sensitivities were calibrated in culture medium using the thermal noise method [34], and the slope of a sample force–distance curve was recorded on a stiff substrate as usual [34] (glass Petri dish in this case). Cantilevers were equilibrated for 30 min in culture medium before calibrations. AFM control measurements in chambers without cells were performed in 2% detergent solution in water (Triton X-100, Sigma-Aldrich Chemie GmbH, München, Germany). No additional CO_2_ supply was used.

### 2.6. Noise Analyses

During approach, cantilever deflections were recorded in a range of 1.5 µm to 1.0 µm above the sample. These curves were corrected for a linear drift, and the remaining noise was analyzed by fast Fourier transformation (Origin 2015 G; OriginLab, Northampton, MA, USA). Time was chosen as the integration variable. A triangular window was used to reduce frequency leakage.

### 2.7. Cell Culture

The keratinocyte cell lines used—keratin I knockout and the corresponding wild-type control—have been described previously [35,36]. Both were cultivated at 5% (v/v) CO_2_ and 32 °C in keratinocyte medium as described previously [37]. This medium contained only a low concentration of 50 µM Ca^2+^ to prevent formation of cell–cell contacts. Chamber bottoms were coated with 20 µg/mL fibronectin (human; Corning) in phosphate-buffered saline (PBS; Thermo Fisher Scientific, Waltham, MA, USA) overnight. Subsequently, 200,000 cells were seeded in each chamber (inner area ~4 cm²). After an adhesion period of 60 min, the medium was replaced by keratinocyte medium supplemented with a higher Ca^2+^ concentration (1.8 mM) to allow epithelial sheet formation. Directly before measurements, which began 17 h after cell seeding, the medium was exchanged again with freshly prepared and thermally equilibrated high Ca^2+^ medium buffered with 25 mM HEPES (4-(2-hydroxyethyl)-1-peperazineethanesulfonic acid; Sigma Aldrich). Measurements were done within a 75 min period after the last buffer exchange.

### 2.8. Light Microscopy

The radius of the microsphere on the cantilever tip was determined with an upright microscope (Axio Imager.M2, Carl Zeiss, Jena, Germany) equipped with a 40×/0.6 NA LD-Achroplan objective (Zeiss). The thicknesses of spin-coated PDMS layers as well as bead patterns for strain calibration were determined with the same upright microscope equipped with a 10×/0.3 NA W N-Achroplan lens (Zeiss). Correction of the optical pathway was done with the refractive index of PDMS n = 1.43. For AFM measurements, cells were imaged using an inverted microscope (Axiovert 200, Carl Zeiss, Jena, Germany) equipped with a 40×/0.6 NA Plan-Neofluar (corr.: 1–1.5 mm) objective (Zeiss) and the recommended equipment for differential interference contrast (DIC) microscopy. Indentation regions were localized with DIC microscopy.

### 2.9. Statistical Analyses

Nonparametric tests were performed. A two-sided Wilcoxon signed rank test was used for matched data pairs, and Mann–Whitney *U* test was used for unpaired data. Significant differences are indicated in the diagrams (not significant (ns) *p* > 0.05, * *p* < 0.05, ** *p* < 0.01, *** *p* < 0.001).

## 3. Results

For the mechanical characterization of strained cells, several challenges had to be met. A biocompatible elastomer system withstanding large mechanical strains had to be established. The stretcher system had to be adapted to the spatial limitations of atomic force microscopy and, finally, the problem of cells moving out of the field of view during stretching had to be solved. Moreover, the pick-up of acoustic noise by the stretched elastomer lamella turned out to be troublesome. In the following sections, we will describe step-by-step how these challenges were overcome.

### 3.1. Enabling Extremely Large Strains in Elastomeric Chambers

In the so-called security belt hypothesis [38], keratins are supposed to act as a mechanical buffer system that protects epithelial tissues against mechanical failure. To test this hypothesis, we applied physiological strains [23] in regimes where reconstituted IFs start to stiffen (20% strain and more [12]). However, in our experience, most silicone elastomer systems fail at lasting or repetitive strains of about 30%–40%. Therefore, we selected a silicone elastomer system that was optimized by the manufacturer for mechanical toughness. To enable chamber molding, we had to extend the possible processing time of the formulation by lowering the process temperature to 6 °C. The resulting chambers were mechanically stable up to several days at a constant, high strain of 100%. However, this material was unphysiologically stiff (370 kPa; epithelial monolayers exhibit a stiffness of about 20 kPa [18]). This, in turn, was solved by overlaying the chamber material with a softer silicone elastomer (50 kPa) that had been extensively used before for cell biological work. Careful control by light microscopy (differential interference contrast, also at high material strain, see below) showed no indications of tearing or other damage of the silicone layer during chamber stretch.

### 3.2. Matching the Stretching Device to an AFM

**Figure 1:**
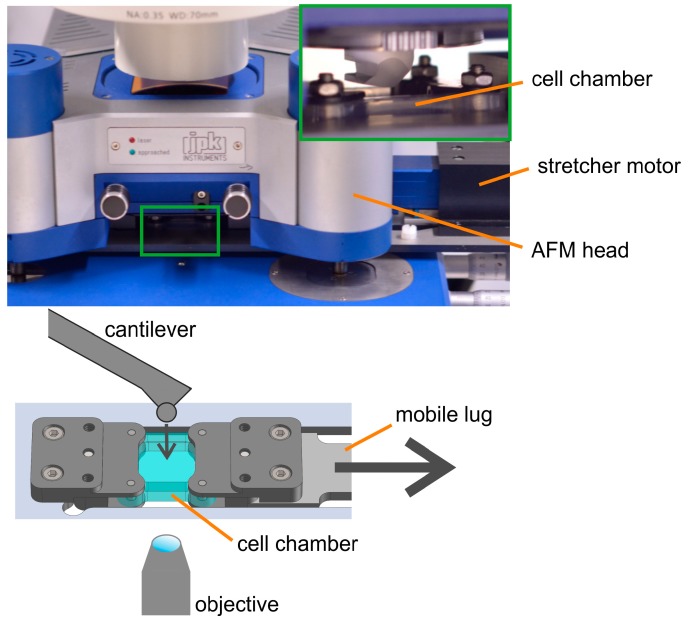
Cells were cultivated in silicone rubber chambers. These were uniaxially stretched by a linear stepper motor connected with a movable lug. Atomic force microscopy (AFM) access is from the top and optical microscopy from the bottom. Photograph of setup (**top**), size 23 cm × 14 cm; zoom-in (green rectangle) size 3.6 cm × 2.3 cm. Sketch of setup (**bottom**) not to scale. Arrows indicate directions of stretch and indentation.

Atomic force microscopes are built extremely compact to minimize sensitivity to external mechanical noise. As a consequence, the space is extremely tight, and it is difficult to fit an additional device under the AFM head. To cope with this, we decided to replace the adjustable sample stage from the microscope table with a new stretcher system with extremely flat chamber holders to fit in the available space. The lug connecting chamber holder and linear drive was elongated to place the bulky stepper motor outside of the AFM head. With these adaptations, the combined setup fitted on the table of our light microscope. Hence, suitable cells and locations could be selected by light microscopy, and samples could be continuously observed optically. A sketch of the combined setup is shown in Figure 1. 

To minimize the impact of spatially varying mechanical properties, we needed to design a setup enabling AFM analyses on the same cells at different strain amplitudes. This way, the externally applied strain would be the only experimental parameter being varied. However, in our setup, i.e., a uniaxial stretcher with one movable lug, areas located next to the immobile lug will stay near to their initial positions, while areas located directly next to the movable lug will be moved by almost the full travel of the motor. We determined these displacements by tracking characteristic bead patterns and found a linear relationship between the distance to the immobile lug and the actual displacement upon stretching. To enable compensation of these displacements, we mounted the stretching device on the moveable translation stage. Thus, we could follow our samples during stretching. 

### 3.3. Setup Characterization

**Figure 2:**
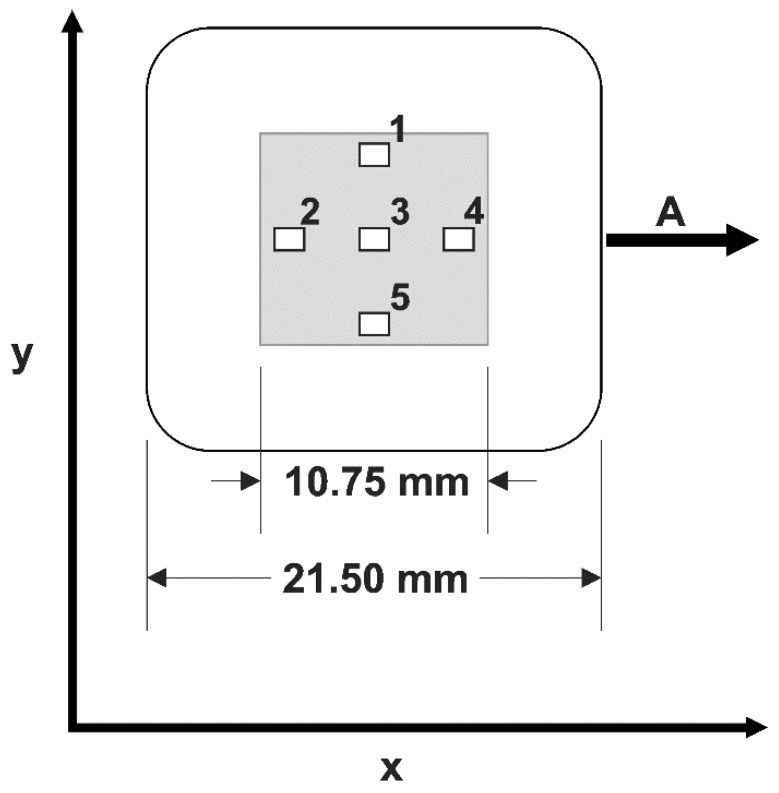
Local strains were determined in five regions (1–5; size 1.40 mm × 1.05 mm) within the inner square of elastic chambers. The chamber was stretched in x direction (A). Sketch to scale. Results of calibration are presented in Table 1.

For further characterization of the cell stretching device, we determined bead displacements during stretch to calibrate homogeneity and magnitude of the local strain. Therefore, we analyzed five different locations distributed on the surface of the elastic chamber, as indicated in Figure 2. Within each of these regions, the local strain was analyzed in more than 30 rectangular templates (33 µm × 263 µm) (Table 1, raw data freely available at the public repository zenodo, see link at end of paper) by the two-step algorithm described above. For both the strain $\varepsilon_{xx}$ in the stretch direction and the shrinkage $\varepsilon_{yy}$ transversal to that, we calculated the transversal shrinkage factor $\kappa =-\varepsilon_{yy}/\varepsilon_{xx}$ (see also Table 1). Throughout the whole procedure, the surface remained flat, as indicated by the focus quality of beads scattered over the surface. Therefore, we did not attempt to measure out-of-plane displacements. The overall change in thickness of the chamber floor can be determined from both the in-plane strains and the fact that soft silicone elastomers deform at constant volume (Poisson ratio of 0.5).

**Figure 3:**
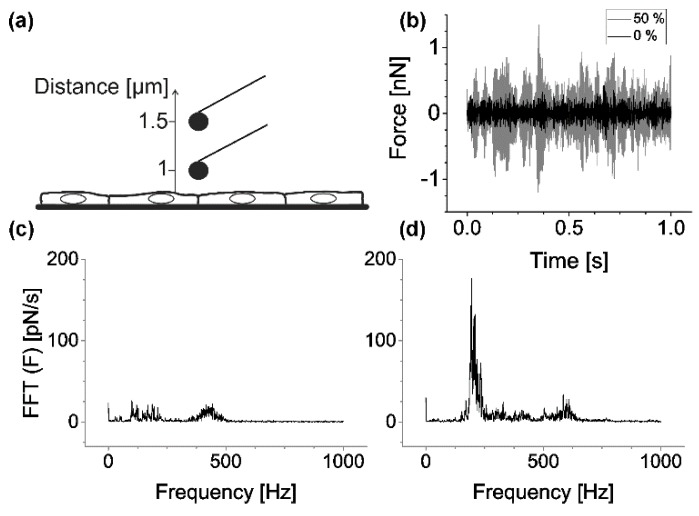
Background noise depends on chamber stretch. (**a**) Principle of mock force–distance curves recorded for noise analyses. AFM cantilever is approaching cell monolayer. (**b**) Exemplary force signals: black, strain 0%; gray, 50%. (**c**) Absolute values of a fast Fourier transformation (FFT) of the force signal at zero strain and (**d**) at 50% strain.

In addition, we analyzed if the stretching device interfered with AFM performance. To this end, we recorded mock force–distance curves during the cantilever approach (raw data can be found in supplement). The cantilever position was determined during an approach from 1.5 µm to 1.0 µm above the sample (speed 0.5 µm/s), the linear trend was removed (cf. Figure 3a), and the remaining noise component was analyzed via fast Fourier transformation as described in Materials and Methods. We found that the overall noise contained a major contribution of unknown origin at about, but not exactly, 200 Hz. Further peaks in the noise spectrum were found at higher frequencies (400 to 600 Hz). Peak positions and heights varied widely between different measurements. Because noise amplitudes increased with strain, meaningful AFM analysis was only possible for strains up to 50% (cf. Figure 3b–d). 

**Figure 4:**
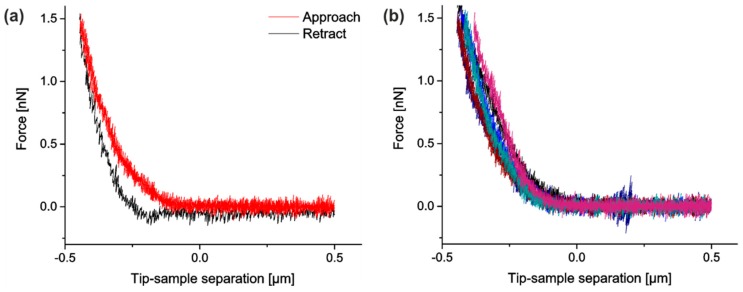
AFM force–distance curves. (**a**) One sample AFM approach (red, color online) and retract (black) cycle recorded above the lamella of a wild-type (WT) cell at 0% stretch. (**b**) Superposition of six AFM force–distance curves recorded successively at the same position above the lamella of a WT cell at 0% strain and intervals of 15 s.

Furthermore, we implemented several measures to reduce experimental noise. In detail, the force–distance curves were smoothed by a moving average filter (width 25 data points corresponding to 6.25 nm or 12.5 ms) before contact points and indentation depths were determined. We also tested other filters for smoothing like a Savitzky–Golay-filter (2nd as well as 4th order, width 200 data points), which gave similar results. Each force–distance measurement was repeated six times. Because reproducibility was very good (cf. Figure 4), we averaged over these indentations. Moreover, the same indenter was used for all measurements. Raw data of all indentations on cells are freely available at the public repository zenodo, see link at end of paper

To test for the possible influence of the soft chamber on indentation experiments, we indented at different strain amplitudes but identical positions on an empty chamber coated with soft silicone elastomer (raw data can be found in supplement). We found very low indentation depths (~75 nm) compared to cells (~500–1000 nm at 1.3 nN; see below). There were no significant differences between indentations at different strain amplitudes.

### 3.4. Strain Stiffening is Reduced in Keratin KO Monolayers

**Figure 5:**
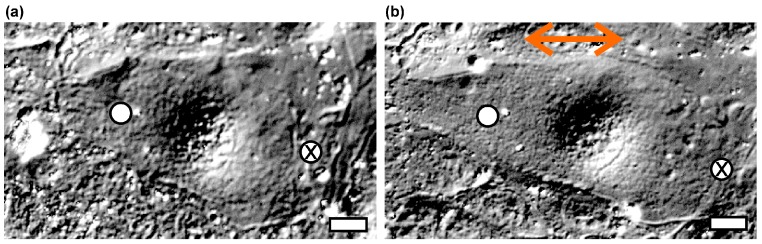
Differential interference contrast (DIC) images of a KtyI^−/−^ cell in relaxed (**a**) and stretched (**b**) state. Strain was oriented horizontally (red double arrow) and amounted to 50%. White dots represent AFM indentations on cell lamellae, crosses on junctions. Scale bars: 20 µm.

To test the influence of keratins on strain stiffening, we compared a complete keratin type I knockout cell line with its wild-type counterpart. Indentations were performed on stable monolayers. Two different regions on each cell were analyzed: above cellular junctions, oriented roughly perpendicular to the strain direction, and above the cell cytoplasm, halfway between the nucleus and regions of cell junctions (see Figure 5, raw data can be found in supplement). For a given cell or cell pair, care was taken to test the same site at each strain level (0%, 25%, and 50%). 

**Figure 6:**
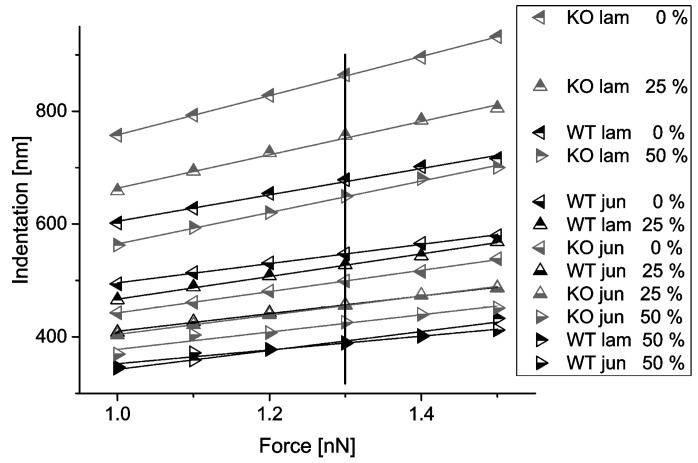
Average indentation depths as function of force for keratin WT and knockout (KO) cells at three different strains. Each symbol represents an average of 14 independent measurements. Scatter similar to Figure 7a,b; not indicated here for the sake of clarity. Slopes were fitted at F_0_ = 1.25 nN of each data set. Vertical bar at 1.3 nN indicates data shown below in Figure 7a,b.

Indentation depths depending on contact forces were plotted from 1.0 to 1.5 nN (see Figure 6). The resulting curves were remarkably linear, and curves corresponding to similar biological conditions (cell type, position) but different strains did not cross.

As the full indentation force of 1.5 nN was not always reached due to fluctuations, statistical analysis was performed on the indentation data taken at 1.3 nN applied force. The indentation depths at 1.3 nN were at least 15% of the overall cell thickness, which was deep enough to probe cytoskeletal structures. These indentation amplitudes corresponded to an average contact area between cell and indenter of 15 µm^2^, that is, only about 0.6% of the cell surface area.

**Figure 7:**
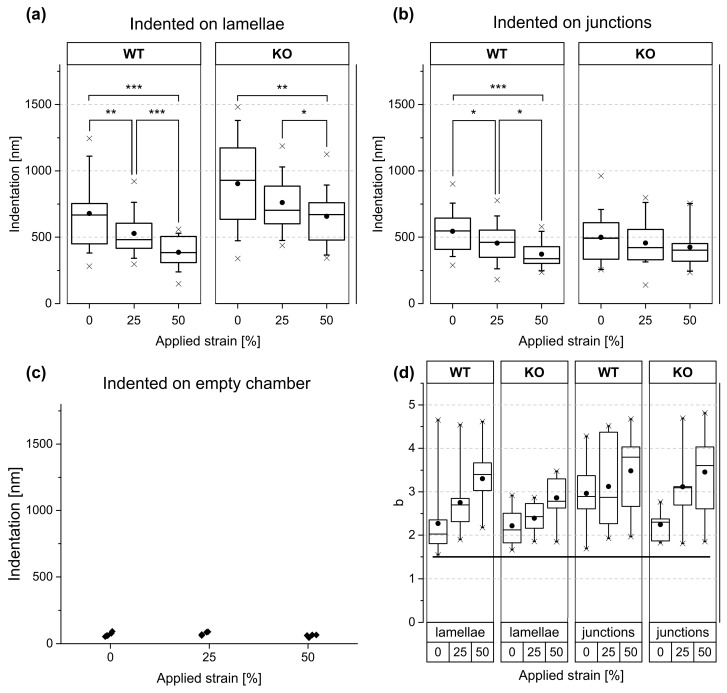
Summary of mechanical analyses of stretched keratinocyte monolayers. Indentations at a force of 1.3 nN on regions of cell lamellae (**a**) and cell–cell junctions (**b**) at different strain amplitudes. Keratin WT cells (left) and KO mutants (right). Sample size was 14 different cells except for KO lamella, where it was 13 (for both lamellae and junctional regions). (**c**) AFM indentation depths on empty chambers. Each dot represents average of four measurements. Significance was tested by a two-sided Wilcoxon signed rank test for matched pairs. (**d**) Exponents of power law (Equation (1)). Bold horizontal line indicates the value required by the Hertz model. Sample size was nine cells for each condition. Boxes represent 50% of the range of values measured, whiskers 80%, and crosses 100%.

The change of indentation depth with increasing strain at constant force is summarized in Figure 7a,b and Table 2. Statistical analyses are presented in Table 3. Over the lamella, keratin-containing (WT) cells displayed a clear reduction of indentation depths for 25% and 50% strain. This dependency was less pronounced for KO cells. Remarkably, stiffening of stretched junctions was significant for WT cells but not for the KO mutant. In addition, we observed that junctional areas of the KO cell line were stiffer than the corresponding lamellae under all stretch conditions. In contrast, the stiffness of WT on lamellae and on junctional areas was similar. Moreover, stiffness of cellular junctions seemed to be independent of the presence of keratin. Cell lamellae were stiffer in the presence of keratin than in its absence at 25% and 50% strain (see Table 2 and Table 3). 

Because the widely used Hertz model resulted in systematic deviations between measurement and fit curve, we used the more general but entirely empiric power law function instead:

(1) $$F={A\delta }^{b}$$

with force F, indentation δ, and the free fit parameters A and b. At rest (0% strain), we obtained a median exponent of 2.03 for WT lamellae and 2.12 for KO lamellae (see Figure 7d). At all other conditions and strains, higher exponents were found. Please note that the Hertz model [1] predicts an exponent of 1.5 and is therefore not compatible with our results. 

Ramms et al. [26] investigated the same cells on glass substrates with AFM. They reported a constant exponent of 2 using isolated cells in the absence of any mechanical stretch. To compare our results at unstrained lamellae to theirs, we also fixed the exponent b at 2. The resulting prefactor A_2_, termed apparent stiffness by Ramms et al., is defined by the following:

(2) $$F=A_{2}\delta^{2}$$

It was determined from the slopes of the indentation versus force plot (Figure 6). For this calculation, linearization of Equation (2) around the central force value (1.25 nN) was used. For WT, we found A_WT_ = 3.6 kN/m^2^ (s.d. 0.62 kN/m^2^) and for KO, A_KO_ = 1.6 kN/m^2^ (s.d. 0.11 kN/m^2^). These values are higher than the ones reported by Ramms et al. but in a similar range.

## 4. Discussion

In the present work, we introduced a new technique that enables precise microrheological analyses of living cells and cell monolayers while subjecting them to prolonged uniaxial strains of large amplitude. Such a deformation mode would occur in human skin, for example, around contracting scar tissue or upon cosmetic surgery. Repeated measurements at identical positions on the same cell but at different strain amplitudes were performed, and the results indicated pronounced strain stiffening. In addition, we were able to distinguish between mechanical responses of cellular junctions and lamellae. Moreover, the mechanical effect of keratin knockout on keratinocytes was clearly detected.

With our system, we could show a nearly one-to-one translation of externally applied strain to local strain as determined by measuring displacements of bead patterns on the chamber bottom. Thus, the strain experienced by cells could be easily adjusted and precisely controlled. We determined the ratio of extension in the strain direction and compression perpendicular to that and obtained a transversal shrinkage factor of κ = 0.20 (s.d. 0.02) for 25% strain and 0.17 (s.d. 0.02) for 50% strain, which is in good agreement with previous findings [22]. Moreover, we found a homogenous strain field across the analyzed area (cf. Table 1). Taken together, all cells within the field of view of the atomic force microscope were subjected to identical substrate deformations.

Next, we performed control experiments on an empty chamber, revealing negligible small indentations in comparison to experiments on cells as well as no measurable influence of chamber strain. Therefore, the influence of the substrates on cell indentation experiments could be excluded.

Indentations on lamellae of keratin KO cells within a monolayer revealed a similar apparent stiffness than was reported for isolated keratinocytes [26]. However, the stiffness of lamellae of WT cells within monolayers was, at average, 70% higher than the value reported earlier [26] for isolated WT cells. Indentations at nuclei of cells in monolayer, done in Homberg et al. [27], revealed 30% less stiffness than those we measured for cell lamellae of keratin WT as well as of KO cells. The same nuclei–lamellae relation was also found in Ramms et al. [26] for isolated cells. Taken together, our data are in reasonable agreement with earlier work on the same cell type. In addition, we observed a tendency of increasing exponents with higher strains (see Figure 7d). This surprising effect deserves further research.

To test our hypothesis that keratin acts as a security belt against high strains in epithelial tissues, we performed AFM indentations on a keratin-free cell model in combination with the matching WT control. We could show strain stiffening on cell lamellae that was more pronounced in the presence of keratins. Furthermore, our results shed new light on the significant but limited influence of IFs on the mechanics at small strains [39]. We found a higher strain stiffening of keratin WT cells than of KO cells, which became even more pronounced at higher stretch amplitudes. This indicated an increased contribution of keratins to the mechanical stiffness at large strain amplitudes. Such an effect had indeed been predicted by Bertaud et al. [40], who used a coarse-grained simulation model. In conclusion, we verified our hypothesis that keratins enhance cellular strain stiffening. 

Intriguingly, in keratin WT cells, junctions showed clear strain stiffening, while this effect was absent in keratin-free cells. Hence, at these large strains, the presence of keratins seems to play a decisive role in force transmission from cellular junctions to the cell body.

Strain stiffening is quite common in biopolymer networks [12,41,42] and has also been observed in living cells [43]. In all these works, strain stiffening was observed in the direction of the strain. Here, we observed stiffening in a direction perpendicular to the stretch, which is much less intuitive. In this context, the work of Vahabi and coworkers [44] is of great interest. They applied both shear and uniaxial strain to collagen as well as fibrinogen networks and measured pronounced strain stiffening in the shear, i.e., normal to stretch direction. These authors explained stretch-induced stiffening by the fact that network extension favors extended, taut polymer configurations with correspondingly fewer bent polymers. As filaments are much stiffer in stretch than in bending, this is a natural explanation for stiffening and could also contribute to the effects observed by us. 

In summary, we introduced a new experimental approach that provides the possibility of local rheological measurements of cells and cell assemblies while varying the amplitude of uniaxial mechanical strain. With this approach, we could pinpoint the role of keratins as a mechanical buffer system against high strains in keratinocyte monolayers.

## Figures and Tables

**Table 1:** Principle strains within the regions indicated in Figure 2.

	Strain	*ε_xx_*	*ε_yy_*	*κ*	n
Position 1	25%	25% (1%)	−6% (0%)	0.22	76
	50%	47% (6%)	−9% (2%)	0.19	
Position 2	25%	24% (1%)	−4% (0%)	0.17	93
	50%	47% (2%)	−7% (0%)	0.15	
Position 3	25%	25% (1%)	−5% (1%)	0.19	94
	50%	47% (2%)	−8% (1%)	0.17	
Position 4	25%	24% (1%)	−4% (1%)	0.18	64
	50%	47% (1%)	−7% (1%)	0.15	
Position 5	25%	25% (1%)	−5% (0%)	0.22	34
	50%	47% (1%)	−9% (0%)	0.19	

Strains (strain: preset strain, *ε_xx_*: measured in stretch direction, *ε_yy_*: perpendicular) were determined in templates as described in Materials and Methods. Given are mean values with standard deviations in parentheses. n denotes the number of templates used in the respective region, and $\kappa =-\varepsilon_{yy}/\varepsilon_{xx}$.

**Table 2:** Dependence of indentation depths on strains.

Location	Cell Type	0% Average [nm]	Quartiles [nm]	n	25% Average [nm]	Quartiles [nm]	n	50% Average [nm]	Quartiles [nm]	n
**Lamella**	**WT**	680	460/740	14	530	420/590	14	390	310/510	14
	**KO**	900	630/1170	13	760	600/880	13	660	480/760	13
**Junction**	**WT**	540	420/630	14	460	350/540	14	370	310/420	14
	**KO**	500	340/600	14	460	340/560	14	430	330/450	14

Indentation depths were analyzed on cells at a force of 1.3 nN. Quartiles: 25% of all data are smaller or equal to the first quartile (left value); 75% of all data are smaller or equal to the third quartile (right value).

**Table 3:** Statistical significances.

Comparison	Lamella Versus Junctions		WT Versus KO	
	WT	KO	Lamellae	Junctions
**0%**	ns	**	ns	ns
**25%**	ns	***	**	ns
**50%**	ns	**	**	ns

Statistical differences between stiffness distributions presented in Table 2 were tested by a Mann–Whitney *U* test for unpaired data (not significant (ns) *p* > 0.05, * *p* < 0.05, ** *p* < 0.01, *** *p* < 0.001). Sample size was 14 different cells except for KO lamella, where it was 13.

## Supplementary Materials

Raw data of force–distance curves and local strains are available online at doi: https://doi.org/10.5281/zenodo.2591184.
